# NephroCAGE—German-Canadian Consortium on AI for Improved Kidney Transplantation Outcome: Protocol for an Algorithm Development and Validation Study

**DOI:** 10.2196/48892

**Published:** 2023-12-22

**Authors:** Matthieu-P Schapranow, Mozhgan Bayat, Aadil Rasheed, Marcel Naik, Verena Graf, Danilo Schmidt, Klemens Budde, Héloïse Cardinal, Ruth Sapir-Pichhadze, Franz Fenninger, Karen Sherwood, Paul Keown, Oliver P Günther, Konstantin D Pandl, Florian Leiser, Scott Thiebes, Ali Sunyaev, Matthias Niemann, Andreas Schimanski, Thomas Klein

**Affiliations:** 1 Hasso Plattner Institute for Digital Engineering University of Potsdam Potsdam Germany; 2 Department of Nephrology and Medical Intensive Care Charité – Universitätsmedizin Berlin Berlin Germany; 3 Geschäftsbereich IT Charité – Universitätsmedizin Berlin Berlin Germany; 4 Research Centre Centre Hospitalier de l'Université de Montréal Montréal, QC Canada; 5 Division of Nephrology and Multi-Organ Transplant Program Department of Medicine and Centre for Outcomes Research and Evaluation Research Institute of the McGill University Health Centre Montréal, QC Canada; 6 Division of Nephrology Department of Medicine University of British Columbia Vancouver, BC Canada; 7 Günther Analytics Vancouver, BC Canada; 8 Department of Economics and Management Karlsruhe Institute of Technology Karlsruhe Germany; 9 PIRCHE AG Research and Development Berlin Germany; 10 PIRCHE AG Berlin Germany

**Keywords:** posttransplant risks, kidney transplantation, federated learning infrastructure, clinical prediction model, donor-recipient matching, multinational transplant data set

## Abstract

**Background:**

Recent advances in hardware and software enabled the use of artificial intelligence (AI) algorithms for analysis of complex data in a wide range of daily-life use cases. We aim to explore the benefits of applying AI to a specific use case in transplant nephrology: risk prediction for severe posttransplant events. For the first time, we combine multinational real-world transplant data, which require specific legal and technical protection measures.

**Objective:**

The German-Canadian NephroCAGE consortium aims to develop and evaluate specific processes, software tools, and methods to (1) combine transplant data of more than 8000 cases over the past decades from leading transplant centers in Germany and Canada, (2) implement specific measures to protect sensitive transplant data, and (3) use multinational data as a foundation for developing high-quality prognostic AI models.

**Methods:**

To protect sensitive transplant data addressing the first and second objectives, we aim to implement a decentralized NephroCAGE federated learning infrastructure upon a private blockchain. Our NephroCAGE federated learning infrastructure enables a switch of paradigms: instead of pooling sensitive data into a central database for analysis, it enables the transfer of clinical prediction models (CPMs) to clinical sites for local data analyses. Thus, sensitive transplant data reside protected in their original sites while the comparable small algorithms are exchanged instead. For our third objective, we will compare the performance of selected AI algorithms, for example, random forest and extreme gradient boosting, as foundation for CPMs to predict severe short- and long-term posttransplant risks, for example, graft failure or mortality. The CPMs will be trained on donor and recipient data from retrospective cohorts of kidney transplant patients.

**Results:**

We have received initial funding for NephroCAGE in February 2021. All clinical partners have applied for and received ethics approval as of 2022. The process of exploration of clinical transplant database for variable extraction has started at all the centers in 2022. In total, 8120 patient records have been retrieved as of August 2023. The development and validation of CPMs is ongoing as of 2023.

**Conclusions:**

For the first time, we will (1) combine kidney transplant data from nephrology centers in Germany and Canada, (2) implement federated learning as a foundation to use such real-world transplant data as a basis for the training of CPMs in a privacy-preserving way, and (3) develop a learning software system to investigate population specifics, for example, to understand population heterogeneity, treatment specificities, and individual impact on selected posttransplant outcomes.

**International Registered Report Identifier (IRRID):**

DERR1-10.2196/48892

## Introduction

End-stage kidney disease is growing globally affecting already up to 800 million (10%) people worldwide. Dialysis or kidney transplantation are current options for renal replacement therapy. There are 100,000 dialysis patients in Germany, and 50,000 in Canada [1,2]. In total, 1992 kidney transplantations in Germany and 1673 kidney transplantations in Canada were performed in 2021 [3,4]. Kidney transplantation is the preferred way of renal replacement therapy because it improves the quality of life for patients, extends life expectancy, and saves resources of the health care system [5-7]. However, there is a shortage in donor organs. As a result, in total 6593 patients in Germany and 3060 in Canada were on a waiting list for a suitable donor kidney in 2021 [4,8]. Despite the advances in medicine and improvement of short-term graft survival rates within the first year, long-term graft survival remains stagnant. It shows an attrition rate of approximately 5% annually after the second year resulting in reinitiation of dialysis or transplantation in approximately 50% of patients after 10 years [2,9]. After transplantation, regular follow-up visits especially in the first year are required to detect and prevent fatal outcomes, for example, infections, kidney rejections, or cancer due to over- or underimmunosuppression. Today, nephrologists lack adequate diagnostic measures for assessing and stratifying the individual patient’s risk for posttransplant outcomes, for example, graft failure or rejection [10]. Early detection of patients at high risk would open an additional prevention path: clinicians would have the opportunity to initiate countermeasures earlier and prevent fatal outcomes.

**Figure 1:**
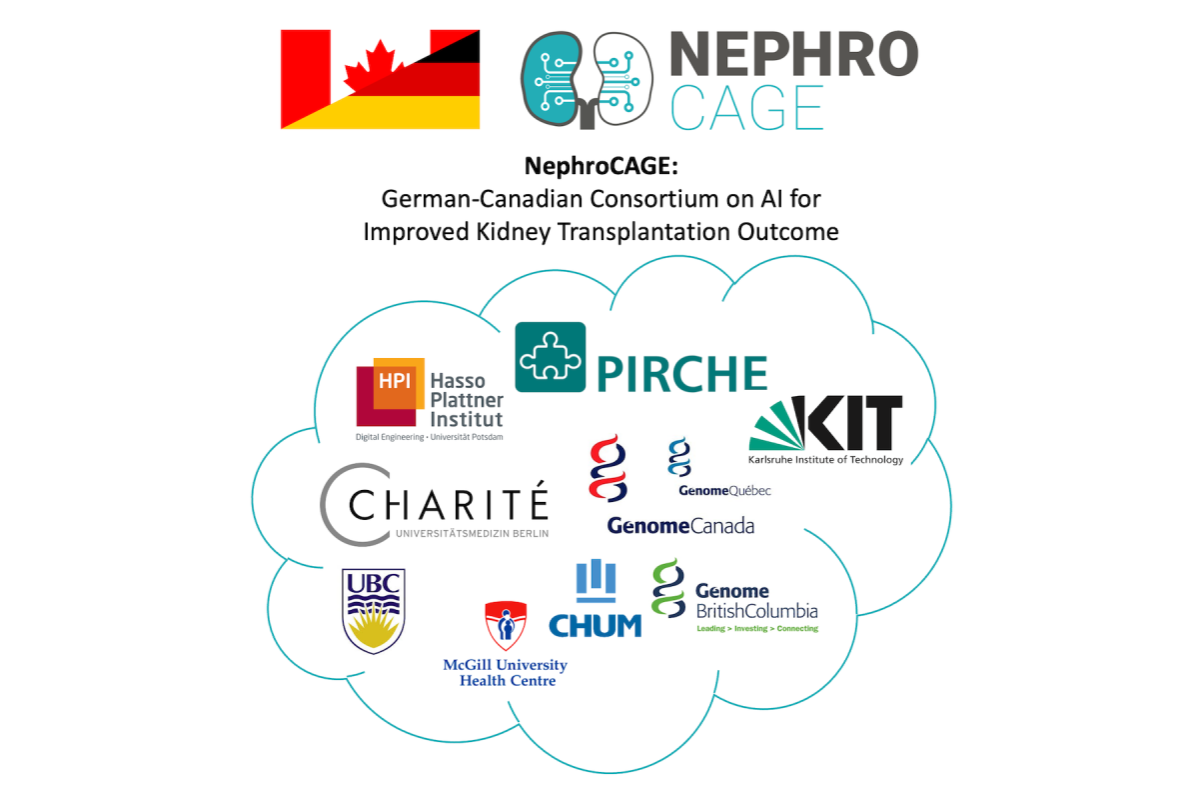
For the first time, the NephroCAGE consortium brings experts from nephrology, academia, and industry on both sides of the Atlantic Ocean together to investigate privacy-preserving ways to enable combination of real-world transplant data from Germany and Canada for design of clinical prediction models to predict the patient-specific probability for severe posttransplant risks.

The NephroCAGE consortium was initiated as a strategic lighthouse project supported by the national governments of Germany and Canada to combine medical and technical expertise to build a real-world demonstrator and evaluate the added value of artificial intelligence (AI) in a very specific medical use case from nephrology. It brings together worldwide excellence from transplant centers, academia, and industry from Germany and Canada to join forces as depicted in Figure 1. In total, 4 major clinical kidney transplant centers from both nations have contributed transplants to form the first-of-its-kind international NephroCAGE data set; it forms a longitudinal database of patient-level data from more than 8000 transplant cases over the past 2 decades. The NephroCAGE data set builds the foundation for detailed retrospective data analysis using AI learning techniques and the development of clinical prediction models (CPMs) for prospective identification of posttransplant outcomes of kidney transplant patients. With the help of the CPM predictions, clinicians are expected to improve the quality of care for future patients with kidney disease in Canada, Germany and worldwide by identifying individual patient risks even earlier than possible today.

Immunological differences play a key role in the development of organ rejection reactions, which might lead to graft failure or even graft loss [11,12]. It has been proven that transplant patients sharing compatible serologic human leukocyte antigen (HLA) types with the organ donor have better outcomes compared to patients with incompatible HLA types [13]. Today, only a subset of HLA genes is considered for matching of immunological factors from donors and recipients. The HLA genes located on chromosome six are responsible for encoding of proteins that fold complex structures, so-called epitopes. Today, it is known that checking for HLA compatibility requires in addition to pure genetic information also the assessment of the protein’s 3D structure, which might trigger immunological response. However, current organ allocation algorithms in productive use include only very limited aspects of HLA compatibility, for example, number of mismatches per HLA, to reduce the immunological risk for graft rejection and donor-specific HLA antibodies [14,15]. Recently, new matching methods have been developed to optimize assessment of organ compatibility between donors and recipients incorporating more functional aspects, for example, on biological level and additional molecular specifics [16-18]. Selected methods showed improved precision in predicting immunological risk, some of them being also relevant for organ allocation [11,17,19,20].

The NephroCAGE consortium aims to show advantages of using molecular compatibility for matching of organ donors and recipients. Therefore, we aim to incorporate the latest research results on B cell and T cell epitope matching as a specific feature of our CPMs. Furthermore, we will investigate the applicability of tree-based machine learning (ML) algorithms such as random forest (RF) and Extreme Gradient Boosting as a foundation for the development of CPMs trained on real-world transplant data in nephrology.

Therefore, access to high-quality real-world data is crucial to train and validate high-quality AI models for clinical use. At the same time, clinical data are considered as highly sensitive data, which typically cannot be exposed for training of AI models. The NephroCAGE consortium as the first of its kind has access to multicenter transplant data from 2 nations for the development of AI-based CPMs. One of our hypotheses is that AI-based CPMs combining both clinical and immunological data will lead to improved detection of patients at high risk for graft loss and rejection. Conversely, we will focus on data from patient follow-ups and immunological data, for example, presence of donor-specific antibodies (DSA) for HLA.

Over the past decades, hospitals and transplant centers have developed individual clinical information systems for management of their transplant patient data. As a result, data are scattered across multiple silos using various data formats, which makes multisite research a complex data management task. Thus, the use of standardized data formats, common data dictionaries, shared terminologies and ontologies, and open application programming interfaces (APIs) are required to facilitate the deployment and integration of innovative AI-based solutions into existing clinical IT systems.

Combining data from multiple clinical data sources for development of CPMs traditionally involves complex data preprocessing steps, for example, data harmonization, data transformation, enable semantic interoperability, legal agreements, and data privacy measures [21]. The use of a federated learning infrastructure (FLI) turns around the paradigm of centralized data storage: it enables transfers of algorithms to the data to perform local data processing, thereby keeping data at its original protected location [22,23]. In a decentralized FLI, network nodes located at collaborating transplant centers communicate on a peer-to-peer basis, that is, all network communication is performed between individual network partners without the need for any central instance. This raises the questions about how access control to the nodes is enforced, how CPMs are trained, and how to exchange CPMs between sites. Recently, distributed ledger technology (DLT) emerged to guarantee immutable transactions between untrusted parties. These transactions are kept in a consistent state through automated, algorithm-based consensus-building mechanisms, which eliminates the need for third-party trust enforcement [24]. The aggregation of models in FLIs, that is, the combination of individual CPM versions from different partners, is well-established for parameter-based AI approaches such as neural networks. However, only limited research investigates the aggregation of tree-based or kernel-based AI approaches, such as RFs or support vector machines, which are relevant for the given nephrology use case.

## Methods

### NephroCAGE Consortium

The founding partners of the German-Canadian NephroCAGE consortium are depicted in Figure 1 and their functions in the consortium are outlined in the following:

- Transplant centers: Charité – Universitätsmedizin Berlin, McGill University Health Centre and Centre Hospitalier de l’Université de Montréal both located in Montréal, Quebec, Canada, and Vancouver General Hospital of the University of British Columbia are internationally known hospitals, each of them with kidney transplant centers covering large patient populations. For example, Charité performs about 200 transplants per year and covers major parts of North-Eastern Germany. Our hospital partners provide access to real-world clinical data, which is key for training and evaluation of CPMs. Furthermore, they provide access to clinical subject-matter experts, drive the clinical focus and lead the development of a clinical demonstrator to evaluate our findings.
- Academia: All university hospital partners are universities affiliated. Furthermore, the Karlsruhe Institute of Technology and the Hasso Plattner Institute for Digital Engineering contribute international expertise in software engineering, AI technology, and digital health. Academic partners contribute through applying the latest AI research findings for building CPMs and to design and implement the NephroCAGE FLI enabling a privacy-preserving way of combining transplant data.
- Industry: PIRCHE AG is an internationally operating company headquartered in Berlin, Germany, having expertise in donor-recipient HLA molecular compatibility assessment. Thus, PIRCHE will work on integration of molecular donor-recipient matching incorporating HLA data from HLA laboratories.

### Ethical Considerations

All methods were carried out in accordance with relevant guidelines and regulations in the participating countries Germany and Canada. The project was approved by the following ethics committees: (1) Charité – Universitätsmedizin Berlin (EA4/104/21) and (2) Research Ethics Board of the McGill University Health Centre and Centre Hospitalier de l’Université de Montréal research center (MP-37-2022-8003). Available data are retrospective data obtained from patients, who gave their informed consent prior to their transplantation for the use of their data for retrospective analyses. All data will be handled in accordance with the corresponding data protection regulations, that is, the European General Data Protection Regulation and the Personal Information Protection and Electronic Documents Act, respectively. Data access was given to clinicians after deidentification only. Prior to the use of data for CPM training, data were in addition pseudonymized to minimize any eventual reidentification risk. There were neither specific compensations nor benefits provided to patients by the NephroCAGE consortium for the use of their data. We use retrospective data, which were gathered during routine care.

### NephroCAGE Data Set

Table 1 provides an overview of the NephroCAGE data set, which consists of more than 8000 transplant patient cases across the past 2 decades with an average age of 51.7 years. Approximately two-thirds of the transplanted patients in our data set are male and one-third are female patients, which aligns with the sex distribution known from related studies [25]. Table 2 provides a summary of available categories of transplant data in our NephroCAGE data set.

Data of transplant patients resides in individual hospital information systems (HIS), for example, laboratory information management system, digital pathology system, or patient management system. For example, patient data are collected during dialysis, at transplantation, and for each of the individual follow-up visits after successful transplantation. Before we can use such data for training of CPMs, each clinical site needs to extract relevant data from their internal source systems and transform them into the common NephroCAGE data schema [26]. Some of the clinical transplant centers have already performed extraction of data from internal sources and its harmonization to a common data schema, for example, due to the presence of a local clinical data warehouse. As a result, local efforts for extraction and harmonization of data are reduced at these sites. Extraction and harmonization of data should be automated to establish a reproducible process to allow the continuous integration of new transplant data into the NephroCAGE data set. Thus, the NephroCAGE data set can also be used for answering future research questions in the clinical domain nephrology.

In the NephroCAGE consortium, we have conducted the following steps to make transplant data available for development of CPMs. All clinical sites defined a study protocol and applied for approval by their individual institutional review boards. It involved the detailed description of required data attributes and what kind of algorithm and models will be implemented on the data, for example, the following details were provided: the goal of the project, research hypotheses, patient cohort description, list of variables, incorporated data protection regulations, exclusion criteria, and methods in particular details about the incorporated AI methods and epitope matching algorithms.

**Figure 2:**
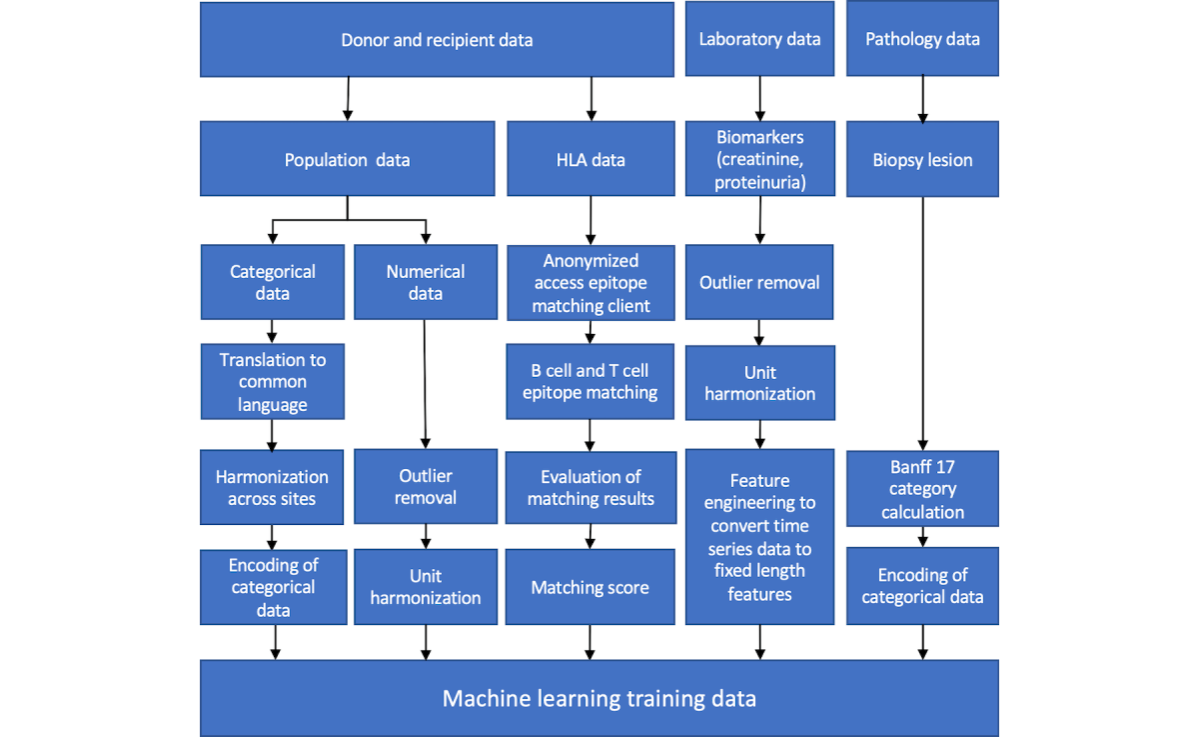
Selected process steps involved in extraction and harmonization of data from NephroCAGE transplant centers to form the NephroCAGE data set. HLA: human leukocyte antigen.

Each clinical transplant center has to identify and extract relevant transplant data from their local HIS. Figure 2 depicts the involved process steps per category of data from top to bottom. After data extraction and format harmonization, the quality of retrieved data needs to be assessed by subject-matter experts. For example, selected cases need to be checked for inconsistencies in reported data. We will only address inconsistencies that occurred as part of the data extraction process. If data are confirmed to be inconsistent in the primary system, we have to exclude the patient case from further processing until these inconsistencies are addressed by the clinical transplant centers. Furthermore, outliers will also be removed from further processing.

**Table 1:** Overview of the NephroCAGE data set: time period, covered years, number of patients, sex ratio, and age distribution. For MUHC^a^ and CHUM^b^ only patients consented Kidney Disease Biorepository–from Birth to Adulthood with first-time kidney transplant were included.

Items	NephroCAGE data set	Charité	UBC^c^	MUHC	CHUM
Period	1998-2020	1998-2020	2008-2018	2012-2019	2011-2019
Duration (years)	23	23	11	8	9
Patients	8067	4742	2510	415	400
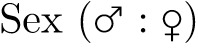 , n (%)	5081 (63%):2986 (37%)	2940 (62%):1802 (38%)	1606 (64%):904 (36%)	279 (67%):136 (33%)	256 (64%):144 (36%)
Age (years), mean (SD)	51*.*7 (14*.*3)	51*.*3 (14*.*0)	51*.*9 (15*.*3)	55*.*6 (12*.*4)	52*.*0 (12*.*8)

^a^MUHC: McGill University Health Centre.

^b^CHUM: Centre Hospitalier de l’Université de Montréal.

^c^UBC: University of British Columbia.

**Table 2:** Categories of transplant data available within the NephroCAGE consortium.

Data category	Description	Selected examples
Recipient data	Measurements of patient data for transplant procedure	Weight, age, sex, HLA^a^ data, transplantation date, type of dialysis, time on dialysis, number of transplantations, delayed graft function, cold ischemia time, death date, DSA^b^, and MFI^c^ of DSA
Donor data	Measurements of donor data for transplant procedure	Weight, age, sex, and HLA data
Laboratory data	Laboratory values, for example, blood and urine	Creatinine, proteinuria (ratio and dip stick), albumin, and CRP^d^
Pathology reports	Medical pathology report	Rejection diagnosis, Banff lesion scores, and Banff diagnostic categories
Clinical notes	Information collected during clinical examination	Diagnoses, symptoms, medical history, physical examination, and written medications
Medications	Information relevant to immunosuppressants	Encoded medication using ATC^e^ codes
Hospitalization data	Details about hospitalizations and clinical assessments	Admission and discharge date, blood pressure, urine volume, pulse rate, and temperature
Follow-up data	Details acquired during regular follow-ups	Weight, blood pressure, urine volume, pulse rate, temperature, and DSA

^a^HLA: human leukocyte antigen.

^b^DSA: donor-Specific antibodies.

^c^MFI: mean fluorescence intensity.

^d^CRP: C-reactive protein.

^e^ATC: anatomical therapeutic chemical classification.

### Improving the Kidney Transplantation Process

**Figure 3:**
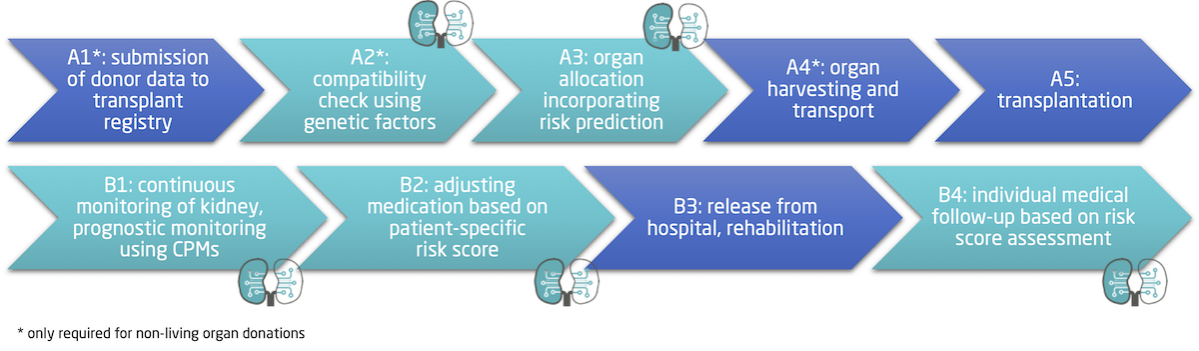
Top: selected clinical process steps taken pretransplant. NephroCAGE aims to enhance the current compatibility assessment between donor and recipient by incorporating genetic testing providing the foundation for advanced organ allocation to minimize the risk of incompatibility of human leukocyte antigen between donor and recipient before organ allocation. Bottom: posttransplant, the function of the graft will be monitored continuously. Comparing data with historical references using the NephroCAGE CPMs enables the definition of a patient-specific risk score to develop clinical end points of interest. As a result, medication and follow-up appointments can be adapted to allow fine-grained monitoring by clinicians. CPM: clinical prediction model.

Figure 3 outlines selected clinical steps in the traditional kidney transplantation process: pre- and posttransplant. If a new organ becomes available via organ donation, its specifics are added to a transplant registry (A1), compatibility checks are conducted (A2), for example, blood group, HLA compatibility, and an allocation decision is taken incorporating donor and recipient specifics (A3). While the organ is retrieved from the donor and transported to the transplant center of the recipient (A4), the recipient is prepared for transplantation (A5). After surgery, the function of the donor organ is closely monitored incorporating common laboratory values (B1) and required medication is set up (B2). After a period of recovery, the patient’s release from the hospital is possible, and rehabilitation can start (B3). Regular follow-up appointments for monitoring of kidney function are required to identify eventual risks for the patient and the graft as early as possible (B4).

We will incorporate B cell and T cell epitope matching for donors and recipients as well as our CPMs to provide new insight for clinicians to decide on a donor kidney allocation in steps A2 and A3. If the decision is made for transplantation, the data from surgery and the recipient’s lab data from the hospital stay will be used as input for the CPMs design in steps B1 and B2. Thus, our CPMs can help to provide patient-specific risk scores to clinicians, for example, to adjust immunosuppressant medication accordingly, and improves continuous posttransplant patient monitoring in step B4.

### Molecular-Genetic Matching

Recently, several algorithms to predict molecular HLA compatibility have been proposed. These algorithms model different immunological pathways of allorecognition. Eplet matching translates HLAs into a set of conformational epitopes, toward which the recipient may form HLA antibodies based on the absence of a corresponding self-epitope [16,17,27,28]. Activation of B cells via conformational epitopes is known to require substantial T cell activation. The model provided by PIRCHE predicts linear T cell epitopes, that may be targeted by the hypervariable T cell receptors. Thus, a combination of specific methods has been shown to further improve histocompatibility prediction [29]. Following this strategy, NephroCAGE aims to combine molecular antibody and T cell epitope matching in a CPM. We aim to implement the molecular matching algorithm as a cloud-based software as a service (SaaS) due to high requirements for storage and computing hardware. The use of a SaaS solution allows flexible control of service quality and user experience. However, submitting genetic data to the SaaS provider may cause conflicts with privacy regulation dependent on local legislation.

Within the NephroCAGE consortium, we aim to develop an open-source command line client for anonymization of HLA data of recipients and donors. For computing the molecular matching scores for donor-recipient pairs, common strategies reducing the resolution of shared information, for example, binning or shuffling, are not applicable given the requirement to obtain exact in silico test results. Therefore, the accurate donor-recipient pair HLA typing will be supplemented by obfuscated HLA data sets. This process considers HLA domain-specific information about haplotype and allele frequencies complicating the identification of individuals in the transmitted data set for the service provider and potential intruders. The molecular matching scores will be correlated to transplant outcome compared to results shown in the literature. Considering these findings as a baseline, we will validate modifications to the prediction pipeline, including a peptide cleavage prediction model.

### Using ML for CPMs

Various related work showed that AI approaches based on medical input data can result in accurate and robust statistical models to predict patient outcomes [30-34]. For example, supervised tree-based ML algorithms, for example, RF and Extreme Gradient Boosting, have shown promising results for classification tasks for posttransplant risks, for example, for graft failure, patient survival, or graft loss within a certain time period [35-40]. However, most of the existing approaches were research-driven and had only limited access to real-world medical data for their work, for example, using the Scientific Registry of Transplant Recipients data set [41]. Loupy et al [42] developed a risk score for kidney transplant recipients in context of a multicenter study making use of multivariable Cox regression analysis to predict graft loss in patients. Among others, they incorporated the following features: estimated glomerular filtration rate, proteinuria, histology, and HLA antibody [42]. Furthermore, recently advanced approaches based on neural networks making use of large proteomic databases occurred for assessment of immunogenicity and probability to generate antibodies [27,43,44].

From a clinical perspective, NephroCAGE aims to prevent the occurrence of adverse posttransplant end points, for example, loss of function, graft failure, and patient death. Nowadays, regular monitoring is applied to help identifying individual risk factors as early as possible. The use of digital patient records in hospitals for more than a decade provides the data foundation for our work because they form a longitudinal database of historic patient cases, medical decisions, therapies, and disease progression. Today, historical case data are only rarely used for current patient care. Our research hypothesis is that the analysis of historical cases can help to derive prognostic predictions for the individual patient risk to reach severe clinical end points. Based on the analysis of patient-specific parameters, a current patient case will be assigned to a group of similar, historic patient cases.

We will use the existing real-world data from historic patient cases from our clinical partners as a foundation for the definition of specific CPMs per clinical end point of interest [45,46]. Together with our clinical experts, we have defined specific CPMs to predict selected short- and long-term posttransplant events, for example, transplant failure and organ rejection. For example, a CPM trained for prediction of graft failure will cover whether this event will happen (yes or no) within 1 year (short-term) or 5 years (long-term). Such a CPM will predict a floating-point probability *p* ∈ [0*,*1], which will be mapped to positive and negative outcome classes. We will minimize the complexity of CPMs by incorporating the principle of data economy, that is, we will only use a minimal set of clinical parameters required to achieve a stable prediction quality. Therefore, we will make us of automatic and manual feature selection approaches to identify most descriptive features for the specific end point from the provided input data set [48-50]. Among others, our CPMs will make use of the following input parameters: recipient and donor data, laboratory values, biopsy results, medication, hospitalization records of the recipient, and HLA compatibility score. Recipient data include sex, weight, height, age at transplantation, end-stage kidney disease, HLA compatibility data, age at graft failure, cause of graft failure, age at death, and many others. Different kinds of follow-up data, for example, weight, and blood pressure, as well as laboratory values, for example, serum creatinine and daily protein-urea. In the case of kidney rejection or failure, a biopsy is performed and analyzed by a pathologist. For interpretation of renal allograft biopsies, Banff classifications are also available in the NephroCAGE data set.

Once a CPM has been trained at a clinical site, it will be shared with other clinical partners in Canada and Germany for validation and continuous training. CPM release versions will be packaged and deployed for execution to individual clinical sites using Docker containers. Thus, comparable small CPMs are exchanged whilst sensitive transplant data do not leave their protected clinical sites.

The current clinical transplantation process outlined in Figure 3 depicts multiple steps where prognostic predictions provided by NephroCAGE CPMs can support clinicians. In the following, we distinguish between pre- and posttransplant use of NephroCAGE CPMs: (1) pretransplant: the use of CPMs prior to transplantation can support more advanced genetic matching of donor and recipients and provide helpful insights for organ allocation; and (2) posttransplant: after transplantation CPMs can also incorporate patient-specific details on the graft function, for example, by including the latest prognosis, which provides a more specific view on the graft. We will focus on binary classifiers predicting short-term (1 year) and long-term (up to 8 years) outcomes post transplantation.

### Patient-Specific Risk Score

The use of patient-specific risk scores is well known in medicine, for example, Glasgow Coma Scale, acute physiology and chronic health evaluation, and sepsis-related organ failure assessment for intensive care patients [51-53]. Risk scores are often used to stratify patients at risk or to predict selected aspects for patient care. They are often designed to use multiple values, for example, latest observational and laboratory values, and combine them toward a single numeric value for a selected clinical outcome. When a patient has a follow-up appointment or encounters any complication, latest data can be incorporated to update the patient-specific risk score.

We aim to define a patient-specific risk score for selected posttransplant events to provide additional insights for nephrologists during posttransplant care. The risk score is a low-dimensional metric representing the overall risk of developing posttransplant complications and will contribute to steps B1, B2, and B4 of the clinical process outlined in Figure 3. It will be calculated by combining the outcome of multiple CPMs and additional patient-specific parameters into a single clinical parameter. Thus, it can support clinicians in continuously monitoring selected kidney function parameters or initiating adequate action as early as possible. Based on the risk score, we will be able to classify patients into low-, medium-, and high-risk patients comparable to a traffic-light schema. Patients classified as high-risk patients will require increased systematic monitoring to prevent complications as early as possible, whereas patients classified as low-risk patients are not expected to develop severe events in the near future. This helps to use the available clinical workforce more efficiently, especially in times of a shortage of skilled labor.

### NephroCAGE FLI

**Figure 4:**
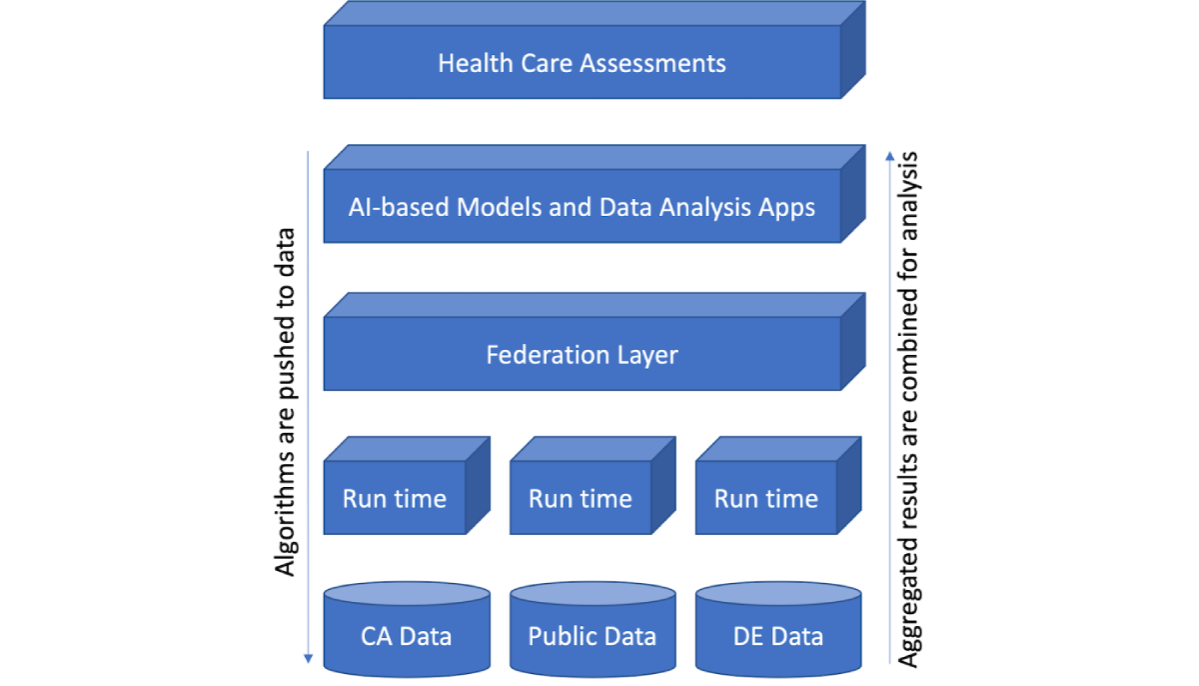
Building blocks of the NephroCAGE FLI. From bottom to top: individual clinical transplant centers from Germany and Canada contribute with more than 2 decades of transplant data enriched by publicly available data, for example, population and research data. Data are only accessible through local runtimes of the NephroCAGE FLI per clinical site, enabling training and sharing of model results whilst keeping data protected. All local FLI runtimes are interconnected through a federation layer, for example, providing access to a data repository for sharing model code and a DLT persistence to store transaction data. The federation layer provides harmonized data access by exposing an API, for example, for training and deployment of AI models and site-spanning data analysis. The results of the CPMs can be assessed by subject-matter experts, for example, to derive patient-specific risk scores for severe posttransplant risks. AI: artificial intelligence; API: app programming interface; CA: Canadian; CPM: clinical prediction model; DE: German; DLT: distributed ledger technology; FLI: federated learning infrastructure.

Figure 4 depicts the building blocks of the NephroCAGE FLI, that is, from bottom to top: local transplant data accessed by local FLI runtimes, a federation layer for exchange of CPMs and data, as well as AI-based model making use of the NephroCAGE FLI to support clinicians in gaining medical insights. Each NephroCAGE clinical partner will join the NephroCAGE FLI by installing and configuring a local NephroCAGE FLI runtime environment on a dedicated host. After transplant data have been extracted from local clinical systems and harmonized, the host running the local FLI runtime environment is granted access to the data. Afterwards, the training of CPMs can be performed on the local data set. Once a stable CPM version becomes available, it will be released for sharing between partners using the NephroCAGE FLI. As a result, pretrained CPMs can be exchanged via the NephroCAGE FLI to facilitate model training even across country borders.

We will use DLT such as an Ethereum blockchain network to have decentralized storage for communicating model updates and code between clinical transplant centers without the need for a dedicated central authority [54]. For support of model training, we will implement an institutional incremental learning approach, where members retrain models one after another and compare different collaborative learning mechanisms with each other [23]. The aggregation of model values is well-established for parameter-based approaches like neural networks. However, only limited research investigates the aggregation of tree-based or kernel-based approaches like RFs or support vector machines, which are relevant for the NephroCAGE use cases.

### Deployment and Integration Into Clinical Information Systems

An expert-facing web app will be developed by the consortium as clinical demonstrator. It will be designed using a representation state transfer (REST) API, which allows integration into existing clinical information systems, for example, T-Base at Charité [55]. The NephroCAGE clinical demonstrator will be used by clinicians to gain insights into patient-specific risk scores including additional information for prospective treatment. Therefore, selected data about individual patients will be exchanged with the CPM to calculate the individual probability for posttransplant risks. As a result, we will provide our CPMs in a local deployable way, that is, the CPMs will be executed by the clinical partners, to preserve privacy of any kind of patient data. Furthermore, the clinical demonstrator will serve as an evaluation platform to identify features that can be intervened in to improve outcome.

To facilitate the integration into existing clinical IT infrastructures, we will follow widely adopted internet protocols (HTTP and HTTPs) and well-known software development paradigms for the development of our CPMs and the clinical demonstrator. Therefore, we will develop the NephroCAGE CPMs as modular software components in Python providing an open API to expose their provided functionality for integration into existing software systems. Thanks to the API of the CPMS, the incorporated programming language and software stack remains transparent for the use of the client app. Through the API a stable software interface describing the required in- or output parameters is available, which will facilitate the integration into our clinical demonstrator and other existing clinical software systems. The REST software paradigm guides the development of web app upon established internet protocols [56]. Hence, RESTful APIs could be designed independent of the existing system with which they will be integrated, making them highly decoupled and modular. We will make use of REST API in our CPM design because it will build on well-established communication protocols for exchange of messages and therefore facilitates the integration into existing clinical IT systems. The execution environment for CPMs requires a diverse set of software dependency to function. Virtualization tools, such as Docker (Docker), can support the packaging of CPM software code, automatically install required software dependencies, and improve software deployment reducing maintenance efforts and hospital IT staff [57]. Thus, our CPMs will be packaged using a Docker container, which reduces deployment and maintenance efforts per clinical site.

## Results

We have received initial funding for NephroCAGE in February 2021. Data owners from all clinical transplant centers have successfully received ethics approval for participating in the project in 2022. The process of exploration of clinical transplant database for variable extraction and harmonization has started at all centers in 2022. Therefore, we have developed software tools to facilitate the extraction, cleansing, and harmonization of relevant transplant data.

For the harmonization of data, we have defined the NephroCAGE data dictionary (DD) together with all clinical partners. The DD covers all attributes per data element, for example, data type, valid data ranges, and harmonized measurement unit. We have 2 types of attributes in the NephroCAGE DD: numerical and categorical. For numerical attributes, we have defined harmonized measurement units, for example, creatinine in µmol/L, age in years, and weight in kg. For categorical data, we have defined all valid categories per attribute, for example, *sex_recipient_* ∈{♀*,*♂}. Some attributes correspond to numerical or categorial attributes depending on the way it was measured. For example, proteinuria can be stored as numerical attribute with the unit mg/day if it was acquired through a 24-hour urine collection or as categorical attribute using the set {+*,*++*,*+++} (+=low, ++=medium, +++=high) if it was acquired using a dipstick quick test.

As of August 2023, a total of 8120 patient records have been retrieved by all clinical partners for use in the NephroCAGE data set as depicted in Table 1. We will calculate the molecular epitope matching score for donors and recipients and extend our data set by it to gain insights in formation of donor-specific antibody formation and impact on the clinical outcome. Subsequently, we will use the NephroCAGE data set to train and validate CPMs for selected posttransplant clinical end points. Locally pretrained CPMs will be retrained and validated by clinical sites through the use of the NephroCAGE FLI. We will focus on ensemble methods to aggregate models trained on individual clinical sites. Setting up the NephroCAGE FLI runtime at each clinical site requires compliance with data compliance regulations of pertaining individual clinical sites and geographies. Our NephroCAGE FLI will facilitate the development of CPMs, enable continuous training at each site without the need for pooling sensitive transplant data in a central database or data warehouse.

## Discussion

### Findings on Using Sensitive Health Data for Development of CPMs

Transplant data used to form the NephroCAGE data set—as any patient data—are very sensitive and requires dedicated protection measures. However, the accessibility of such data is crucial to enable training of CPMs with high prognostic value. Running a multinational project requiring access to data from different geographic jurisdictions is complex, for example, due to compliance with individual data protection regulations and variety of data formats and semantic meaning. The NephroCAGE consortium develops the privacy-preserving NephroCAGE FLI to comply with the regulations of Canada and Germany. Furthermore, the NephroCAGE data set is unique in its size, details, and longitudinal completeness. As a result, we believe that the NephroCAGE data set can also serve future research as a profound foundation. For example, we plan to use the NephroCAGE data set to analyze the impact of demographics and health care systems on the posttransplant outcomes in Germany and Canada.

The complexity of transplant data stems from its multiple attributes originating from different sources. It contains longitudinal data about clinical events typically occurring years or decades after transplantation and comorbidities related to the underlying disease, for example, creatinine from laboratories and hospitalization data from HISs. Data extraction and harmonization across partners is one of the most challenging parts of our project, because each center has its individual IT infrastructure to store transplant data. Due to our federated setup, we do not make use of a central data warehouse. Therefore, harmonizing transplant data before developing CPMs is more crucial than in a traditional data warehouse setup. Thus, we will perform specific data preprocessing activities, for example, harmonizing categorical variables with the same name and units and checking numerical variables for outliers.

We will work together with nephrologists and clinical experts to identify clinical end points of interest as basis for CPM development. We will start to investigate the applicability of tree-based models and artificial neural networks as ML methods based on our literature review, because tree-based methods perform better on imbalanced tabular data than neural networks. Furthermore, tree-based methods show better explainability by providing explicit feature importance. Finally, an imbalance of data in kidney transplantation might lead to over-fitting for the majority classes. Consequently, we will apply resampling methods to reduce this effect [58-60].

Genomic data for the HLA compatibility algorithm are among the most sensitive transplant data. Therefore, we aim to implement an anonymization client so that the HLA is not susceptible to various privacy threats. Today, only 5 genetic loci from HLA-A, -B, and -DR are used by Eurotransplant’s graft allocation algorithm despite a total of 11 clinically relevant HLA loci being associated with immunologic risk [15]. Molecular matching, such as PIRCHE, has been shown to provide additional value to serologic HLA matching in assessing risk of developing donor-specific HLA antibodies, thus having the potential to improve long-term transplant outcomes. Furthermore, donor-specific antibodies are a major factor for rejection that deteriorate organ function and result in graft loss. Although we will use the NephroCAGE FLI to share CPMs between partners, still concerns remain about the privacy of patient data in the case of backtracking the ML model coefficient into individual patient outcomes. Therefore, we will incorporate only deidentified data as basis for CPM training, that is, anonymization of sensitive attributes such as follow-up visit dates, surgery date, and birth date.

Furthermore, the prediction results of CPMs alone are not sufficient to fully explore the reasons. Therefore, we will add additional information to the results, which will enable clinicians to perform informed decision-making, for example, most important features used by the model or details about the specific subtree the individual is assigned to. Thus, clinicians will be enabled to assess the provided prediction and also provide feedback about their final decision, which might be different from the prediction. Thus, we trust that clinicians can incorporate additional insights provided by CPMs into their decision-making process, but still remain the final and human decision maker responsible for clinical treatment decision.

### Limitations

We use observational retrospective data over different time periods and different centers. For example, the majority of transplant data contributed to the NephroCAGE data set is provided by 1 center as outlined in Table 1. Furthermore, transplant data from individual centers was collected across different time periods, thus they might differ due to advances in medical practice. This limits data integration from all centers, including preprocessing and modeling of the data in a similar fashion. Different times and eras of transplantation may infer time bias in the model. Different center practices and demographics may be challenging to interpret and might influence the prediction of CPMs. Therefore, CPMs will show the differential importance of features on the desired clinical outcome. However, clinically actionable features must be determined as certain features cannot be changed, for example, the donor’s age. For some clinically relevant factors, interventions must be found, and randomized controlled trials must be established to find appropriate therapy. By diminishing worse outcomes, training and improvement of the CPM may change over time, resulting in inferior model performance, thus deteriorating identifying patients at risk. All the hospitals represent independent cohort in our federated learning setup and hence a particular attention needs to be given in the order of learning to be done on these data set [61].

### Conclusions

We have introduced the overall goals of our German-Canadian NephroCAGE consortium, addressing multiple challenges in implementing latest federated learning methods to enable privacy-preserving training of CPMs using real-world transplant data from transplant centers in Germany and Canada. We have highlighted the need to perform data harmonization and develop automated data extraction pipelines per transplant center to ensure reproducibility and scalability of the developed CPMs. Our NephroCAGE FLI will be used for privacy-preserved training and exchange of CPMs, which incorporate data from various hospitals. By including molecular epitope matching into our CPM, we aim to gain insights on donor and recipient HLA-matching beyond the current standard of care. The goal of our CPMs is to support clinicians by identifying severe posttransplant risks as early as possible for individual transplant patients. We aim to develop a clinical demonstrator, which will be used for evaluation of CPMs in a clinical setting. The CPM needs to be verified in randomized clinical studies and evaluated to assess factors that can be used for treatment and modification to improve outcomes. This will especially be achieved if more transplant centers join the consortium and share their transplant data via the NephroCAGE FLI.

## Abbreviations

AI: artificial intelligence
API: application programming interface
CPM: clinical prediction model
DD: data dictionary
DLT: distributed ledger technology
FLI: federated learning infrastructure
HIS: hospital information system
HLA: human leukocyte antigen
ML: machine learning
REST: representation state transfer
RF: random forest
SaaS: software as a service

## Data Availability

The NephroCAGE data set is not publicly available. However, detailed information on the data dictionary is available from the authors on reasonable request and will be released to the project website [62].
